# Working with Senior Residents: How to get past “You’re doing great!”

**DOI:** 10.21980/J8D93J

**Published:** 2021-10-15

**Authors:** Caitlin Schrepel, Douglas Franzen, Jamie R Shandro

**Affiliations:** * University of Washington, Department of Emergency Medicine, Seattle, WA

## Abstract

**Audience:**

This content is intended for emergency medicine faculty.

**Introduction:**

Faculty at our institution noted that it can be easy to identify and address the knowledge gaps of junior learners. However, they often find different skills are needed when precepting senior residents, a sentiment shared by faculty at other institutions.^1^ To foster the skills needed for lifelong learning and deliberate practice, it is crucial that educators find methods to effectively assess the skills of senior residents and provide them with continued feedback.^2^ The goal of this faculty development session is to outline methods educators can use with senior residents to support their autonomy and independence while exploring their clinical reasoning, pushing them outside of their comfort zone, and promoting reflective and deliberate practice.^2^–^9^

**Educational Objectives:**

By the end of the lecture, all faculty will be able to 1) describe how to use the Aunt Minnie method of precepting for senior residents, 2) list examples of ways in which they can probe the clinical reasoning of senior residents, 3) explain how to use reflective feedback techniques with senior residents, and 4) report use of the techniques discussed during this session when precepting senior residents in the emergency department.

**Educational Methods:**

This content is presented as a PowerPoint lecture with subsequent discussion

**Research Methods:**

A post-session survey was distributed to allow participants to evaluate the session. This survey was sent four months after the session to gauge how often participants were using the covered techniques on their clinical shifts in the interim.

**Results:**

The majority of survey respondents reported that they initially felt that precepting senior learners was “somewhat difficult” and that they found this session “valuable” in helping them address this challenge. Faculty reported using all of the techniques discussed in this session during their clinical shifts, but were more likely to use methods to promote clinical reasoning or reflective feedback than the Aunt Minnie method of precepting. Overall, respondents reported learning new skills during this exercise and appreciated the format which allowed them to share their own experiences of working with the senior residents and brainstorm techniques that might be useful beyond those discussed by the instructors.

**Discussion:**

This faculty development session successfully introduced emergency medicine faculty to techniques that can be used to improve feedback and assessment of senior residents. The lecture format allowed for efficient transmission of material, while several group discussions and a role-play activity allowed for integration of material and sharing of experiences. Overall this exercise was a success with faculty reporting use of several of the techniques discussed. In future iterations, it may be beneficial to integrate more role-play activities to allow participants to practice more of the skills learned in a simulated environment prior to implementing on shifts with learners.

**Topics:**

Feedback, deliberate practice, clinical reasoning, precepting.

## USER GUIDE

**Table:** 

List of Resources:	
Abstract	7
User Guide	9
Wilderness Explorers The GAME	11
Appen	12

**Learner Audience:**

Faculty

**Time Required for Implementation:** 45 minutes

**Recommended number of learners per instructor:** This lecture works best when limited to 8 learners per instructor in order to facilitate group discussion. However, it could be given to larger groups with the use of breakout sessions.

**Topics:**

Feedback, deliberate practice, clinical reasoning, precepting.

**Objectives:**

By the end of the lecture, all faculty will be able to:

1. Describe how to use the Aunt Minnie method of precepting for senior residents
2. List examples of ways in which they can probe the clinical reasoning of senior residents
3. Explain how to use reflective feedback techniques with senior residents
4. Report the use of the techniques discussed when precepting senior residents

### Linked objectives and methods

Junior learners in the emergency department have much to learn, and the gaps in their knowledge are often easy to identify and address. However, it can be challenging to identify areas for improvement for competent senior residents. How do we continue to actively educate trainees as they become more advanced? How do we help them continue to improve when they seem to be “doing fine?” The goal of this faculty development session was to outline methods educators can use with senior learners to support their autonomy and independence while exploring their clinical reasoning, pushing them outside of their comfort zone, and promoting reflective and deliberate practice. 2–9 This faculty development session utilizes several learning techniques to efficiently transmit information while engaging the audience. The lecture format allows for the dissemination of information to an audience of busy faculty, who often do not have time to prepare for sessions. The integration of group discussions, sharing, and role play engages the audience and gives them an opportunity to explain and demonstrate how to use the techniques discussed. The session itself gives the participants the opportunity to demonstrate their ability to meet objectives 1–3, while a follow-up survey allows for faculty to report whether they have met objective four while working clinical shifts with senior learners.

### Recommended pre-reading for instructor

- Cunningham AS, Blatt SD, Fuller PG, et al. The art of precepting: Socrates or Aunt Minnie? *Arch Pediatr Adolesc Med*. 1999;153(2):114–116.
- Lim FA. Questioning: a teaching strategy to foster clinical thinking and reasoning. *Nurse Educ*. 2011;36(2):52–53.
- Ericsson KA. Deliberate practice and acquisition of expert performance: a general overview. *Acad Emerg Med*. 2008 Nov;15(11):988–94 Epub 2008 Sep 5. PMID: 18778378. doi: 10.1111/j.1553-2712.2008.00227.x
- Fainstad T, McClintock AA, Van der Ridder MJ, et al. Feedback Can Be Less Stressful: Medical Trainee Perceptions of Using the Prepare to ADAPT (Ask-Discuss-Ask-Plan Together) Framework. *Cureus*. 2018;10(12):e3718.

### Results and tips for successful implementation

Five out of the nine faculty surveyed completed the survey after the session. The majority of survey respondents (80%) reported that they initially felt that precepting senior learners was “somewhat difficult” and that they found this session “valuable” in helping them address this challenge. The most common technique faculty reported using following this session was probing for clinical reasoning (80%), followed by reflective feedback (40%) and Aunt Minnie (20%). Overall, respondents reported learning new skills during this exercise and appreciated the format. In particular, they appreciated the time allotted for group discussion, which allowed for faculty to share their own experiences of working with the senior residents and brainstorm techniques that might be useful beyond those discussed by the instructors.

This faculty development exercise can be done in a single session and takes about 45 minutes to complete. To prepare, we recommend that instructors read the suggested reading at least a few days in advance of the session if they are not already familiar with the topics discussed (ie, deliberate practice, Aunt Minnie, clinical reasoning, etc.). It is also important that instructors familiarize themselves with the instructor notes and adapt them as needed. The role-play exercise can be done in several ways. Two instructors can role-play together to demonstrate the use of the techniques, or you can choose to have the audience role play with each other. It is important to decide on how you will do this prior to the session and plan accordingly. Following our session, we distributed a follow-up survey (Appendix A) to gauge the usefulness of the session and to determine which techniques, if any, they started using following the session. Participants reported using the technique of probing clinical reasoning most frequently, followed by the use of reflective feedback and precepting using the Aunt Minnie method.

### Technology necessary

You will need a computer that can run PowerPoint. Because there is a video included in the lecture, it will also be necessary to ensure sound can play over speakers and that the computer is connected to the Internet.

### Assessment (optional)

A post-session evaluation was sent to all participants (attached).

## Supplementary Information



## Figures and Tables

**Figure:**
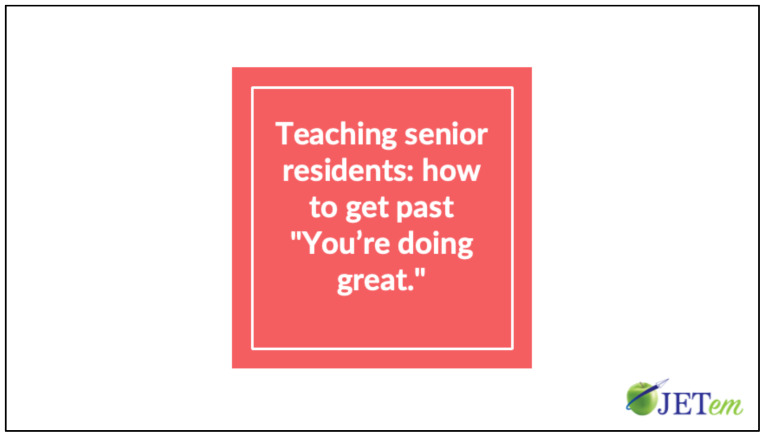
Please see associated video file

## LEARNER MATERIALS

### Appendix A Assessment Survey

1. How challenging do you find it to provide meaningful feedback to competent senior residents on shift?
  **Table:**
  Easy	Somewhat easy	Neither easy nor difficult	Somewhat difficult	Difficult
2. How pertinent was this faculty development session to your practice?
  **Table:**
  Not at all valuable	Not so valuable	Somewhat valuable	Very valuable	Extremely valuable
3. How much did you learn in this session?
  **Table:**
  None at all	A little	A moderate amount	A lot	A great deal
  If you learned new information, what was it that you learned?
4. Which new tools or approaches have you used as a result of this session?
  Aunt Minnie presentations
  Probing for clinical reasoning
  Reflective feedback
  None of the above
5. Which, if any, additional methods do you use when working with senior learners?
6. What suggestions do you have for improving this session in the future?
