# Supplementary material for: Working with Senior Residents: How to get past “You’re doing great!”
Source: J Educ Teach Emerg Med. 2021 Oct 15;6(4):L7–L12. doi: 10.21980/J8D93J (PMC10332739; doi:10.21980/J8D93J)

## Slide 1
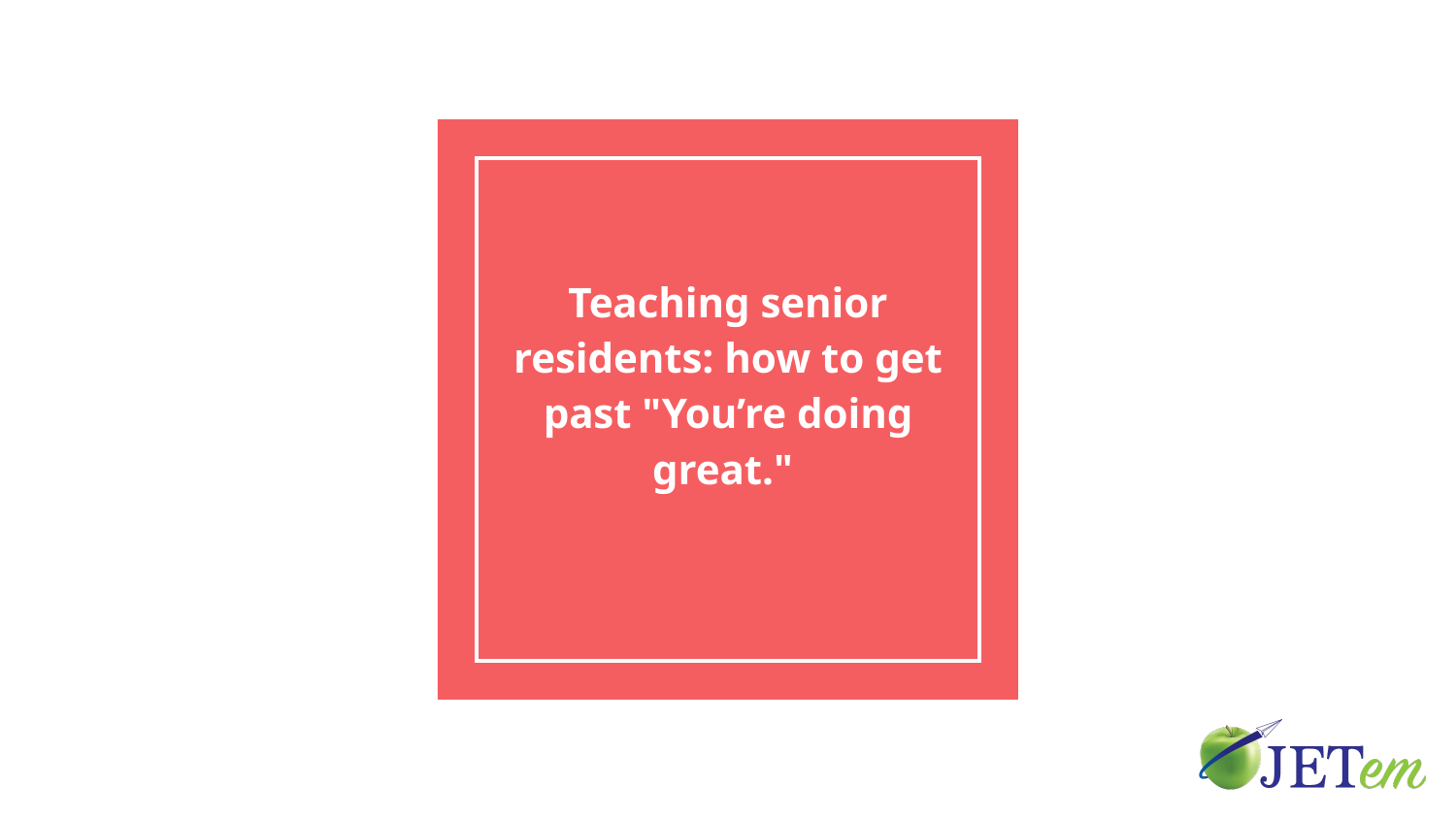

# Teaching senior residents: how to get past "You’re doing great."

## Slide 2
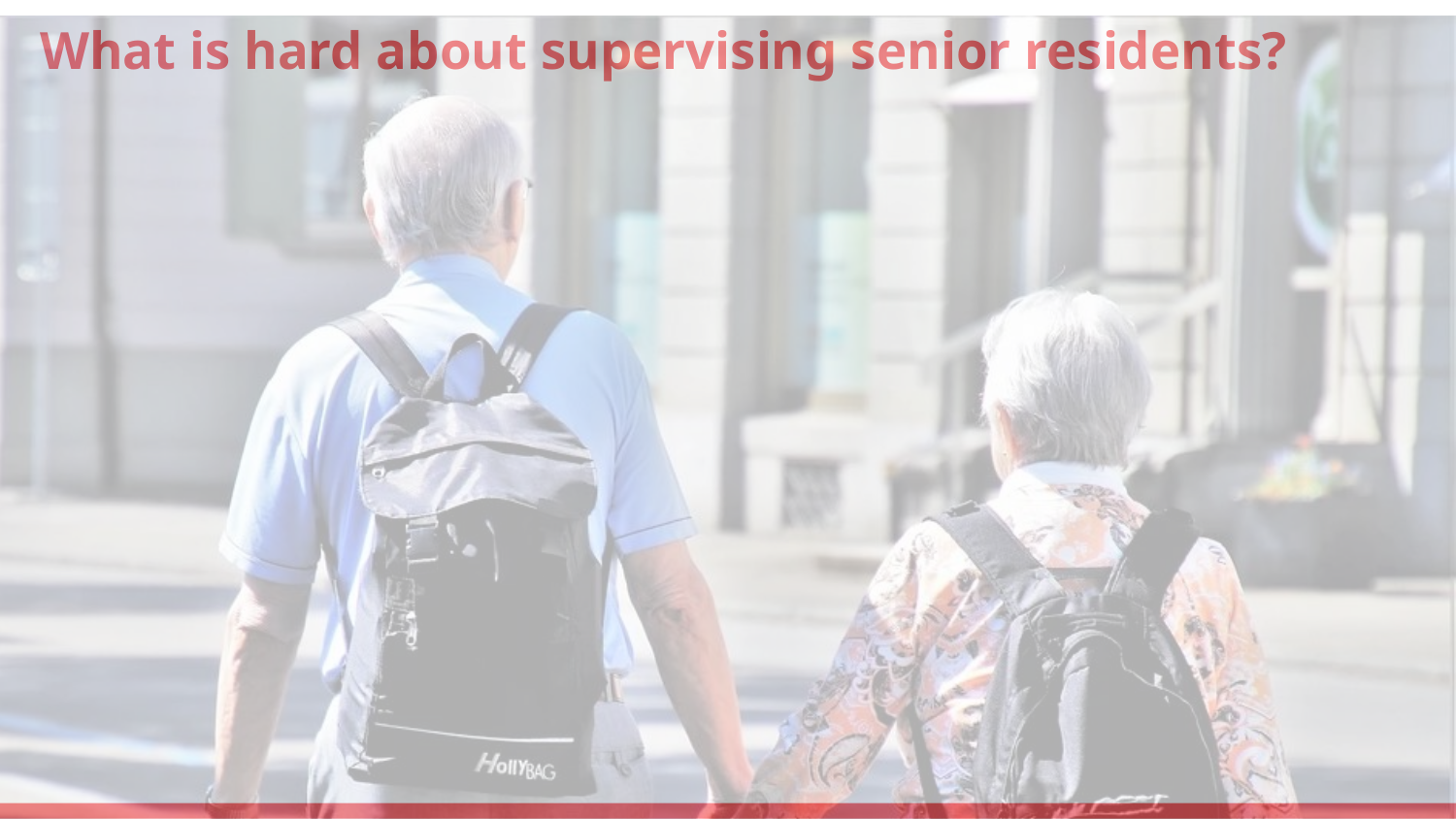

# What is hard about supervising senior residents?

## Slide 3
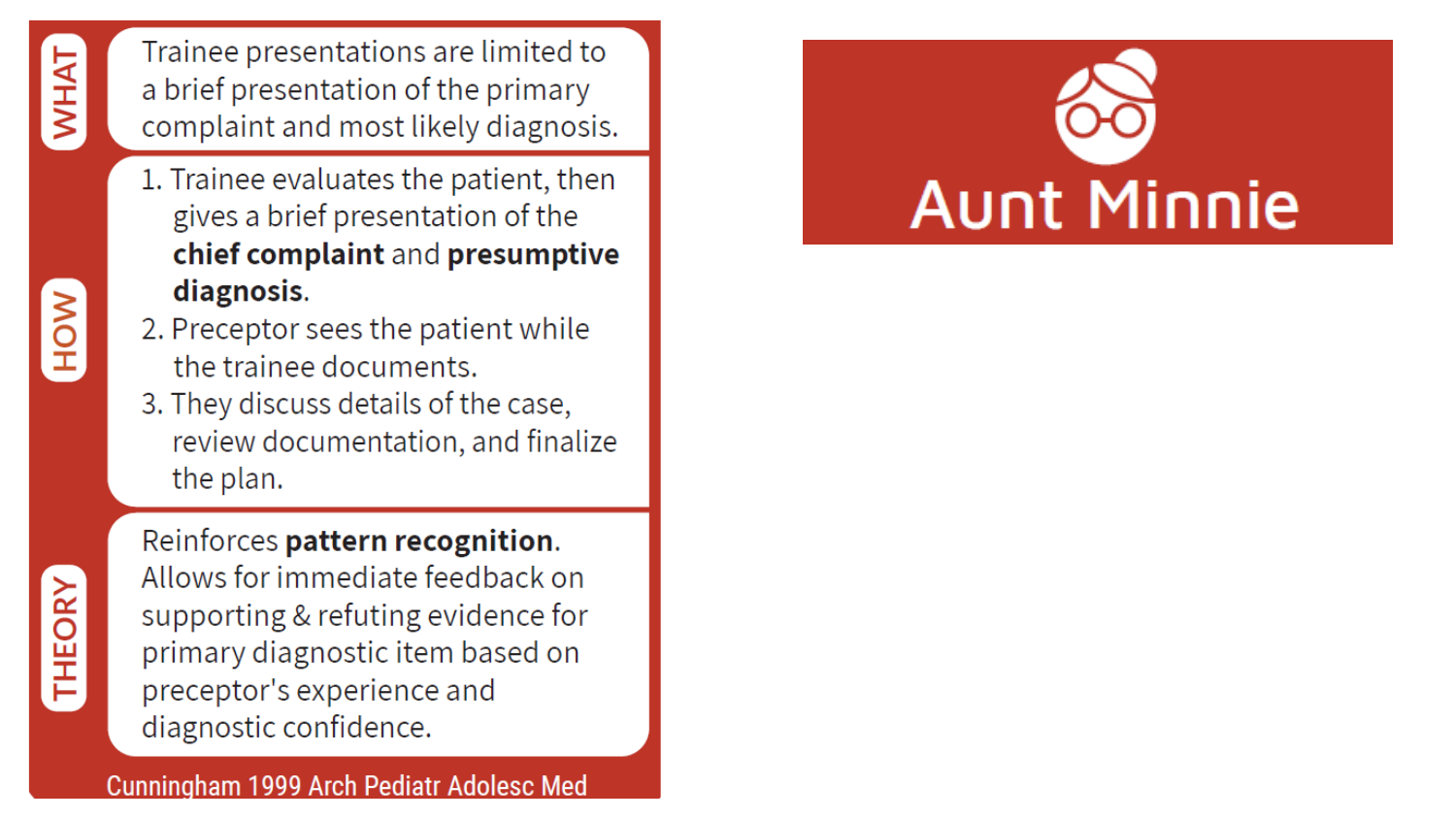

## Slide 4
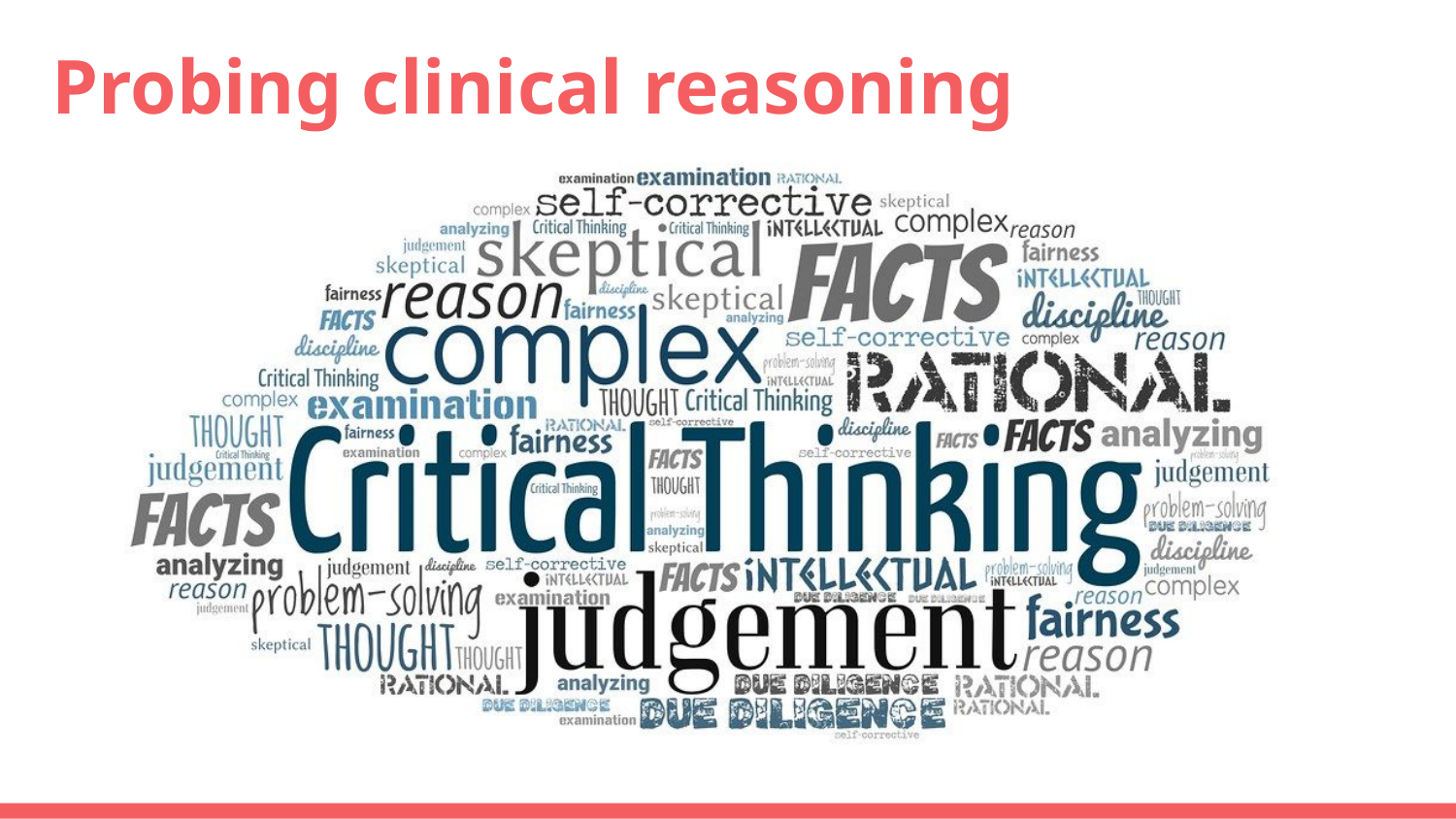

# Probing clinical reasoning

## Slide 5
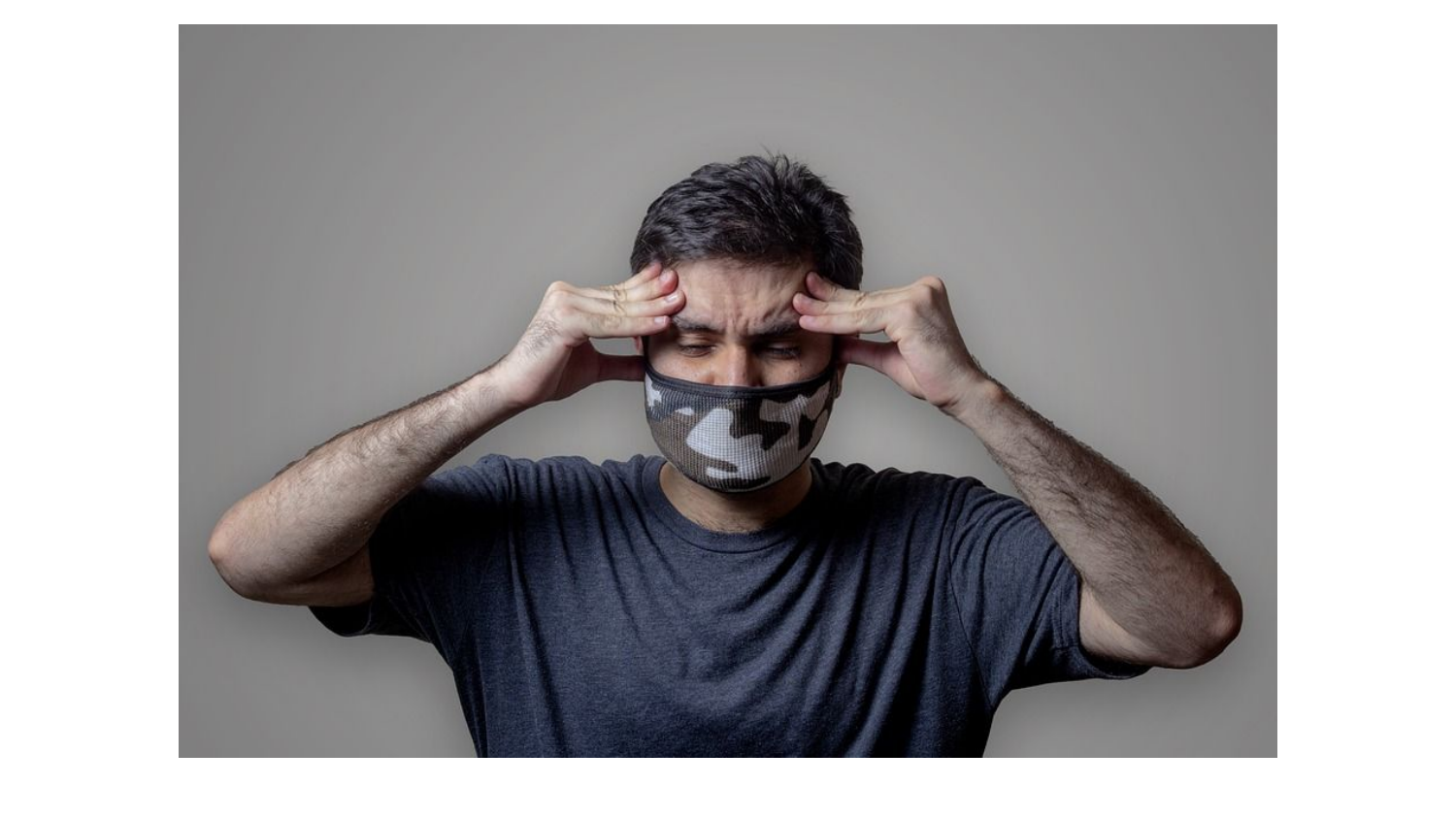

## Slide 6
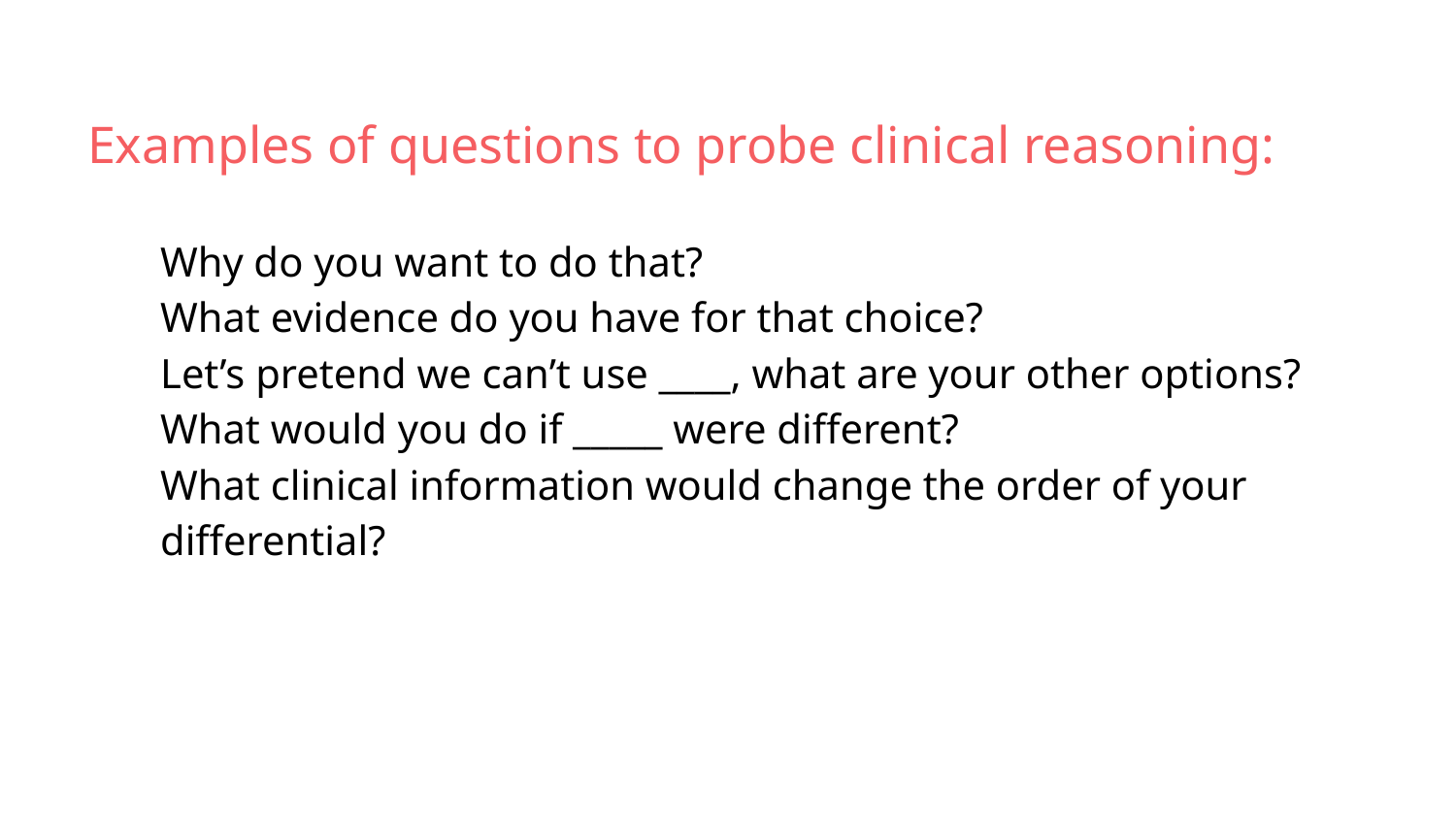

Examples of questions to probe clinical reasoning:
Why do you want to do that?
What evidence do you have for that choice?
Let’s pretend we can’t use ____, what are your other options?
What would you do if _____ were different?
What clinical information would change the order of your differential?

## Slide 7
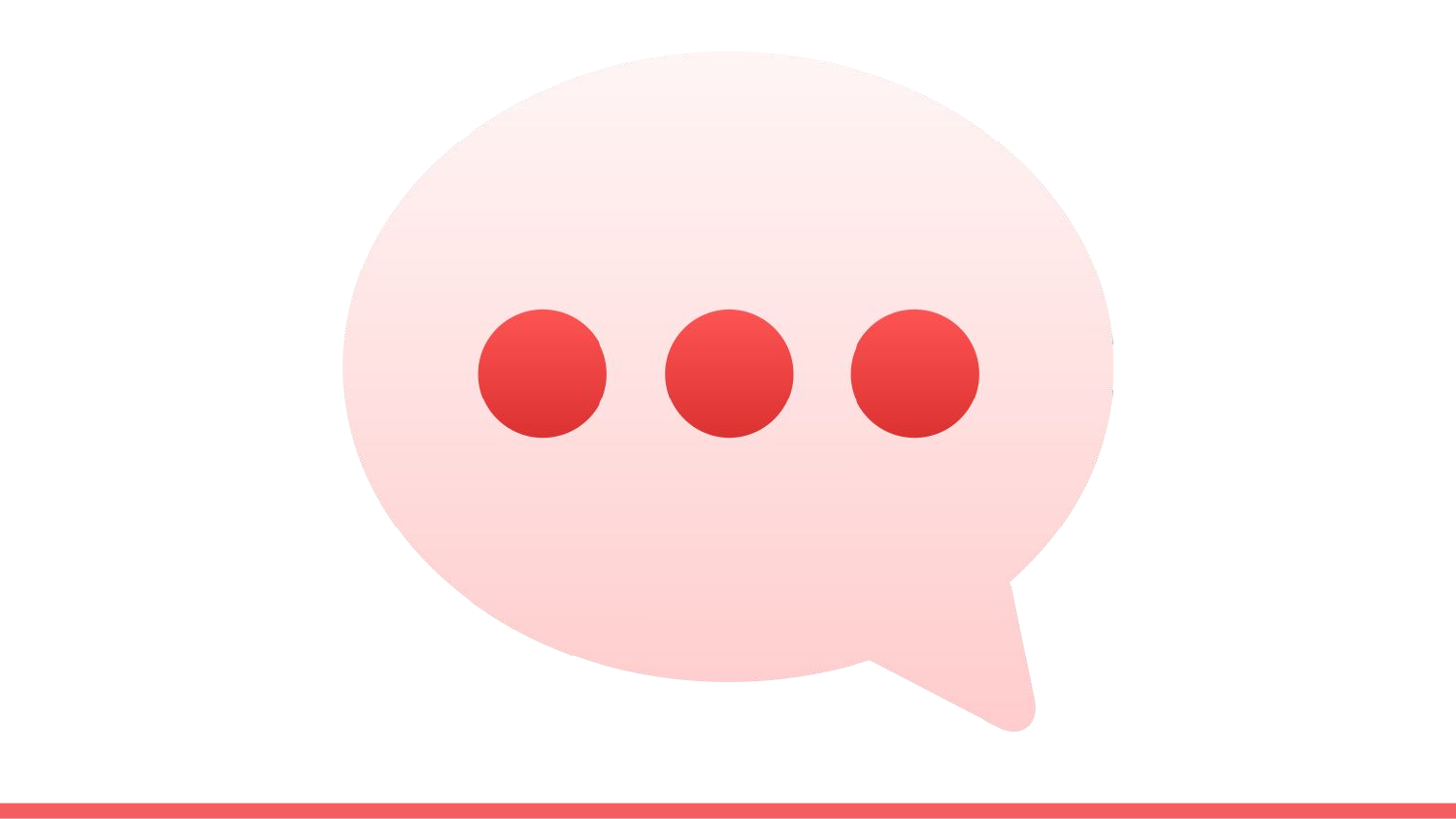

## Slide 8
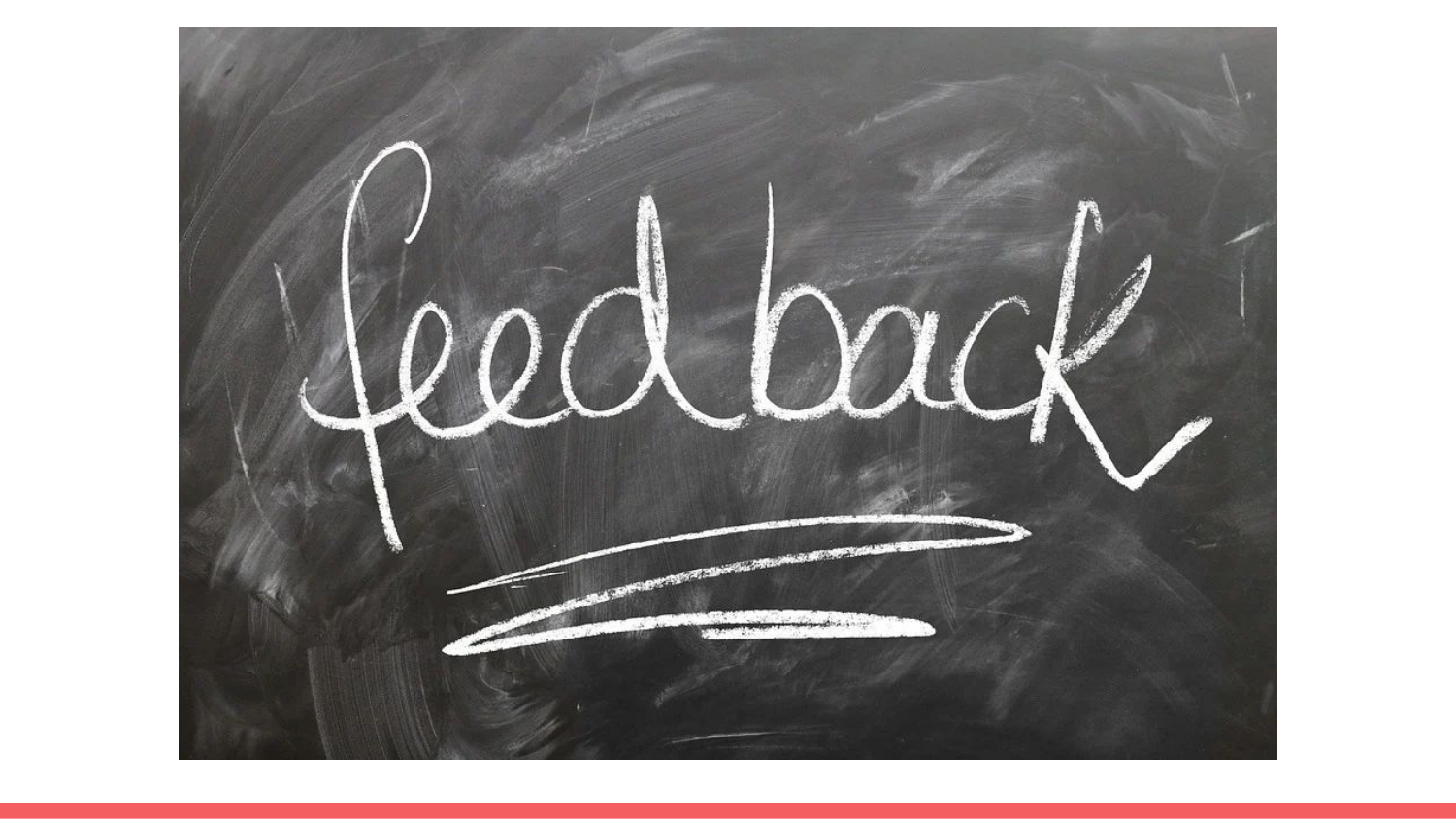

## Slide 9
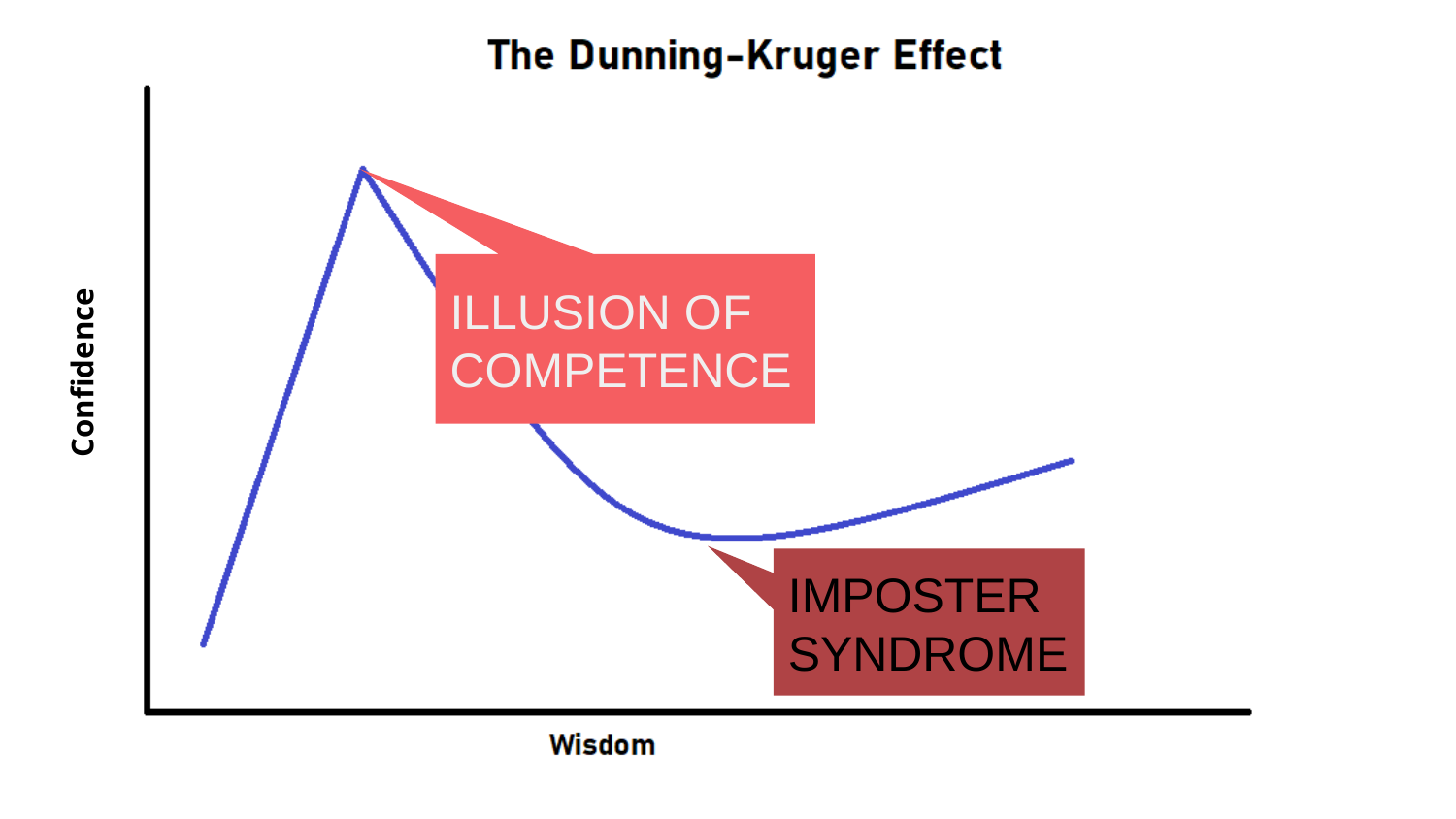

ILLUSION OF COMPETENCE
Confidence
IMPOSTER SYNDROME

## Slide 10
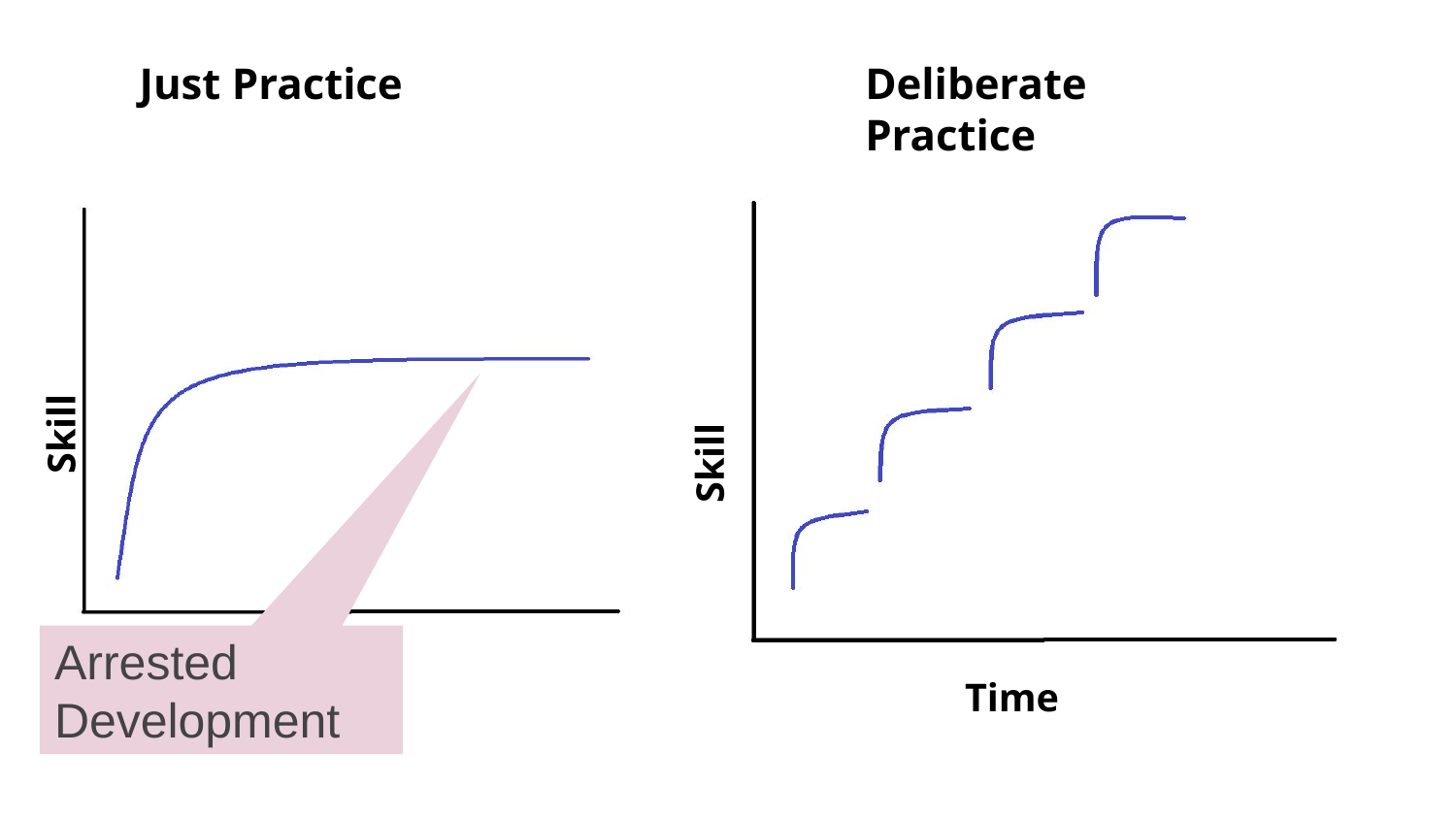

Just Practice
Deliberate Practice
Skill
Skill
Arrested Development
Time
Time

## Slide 11
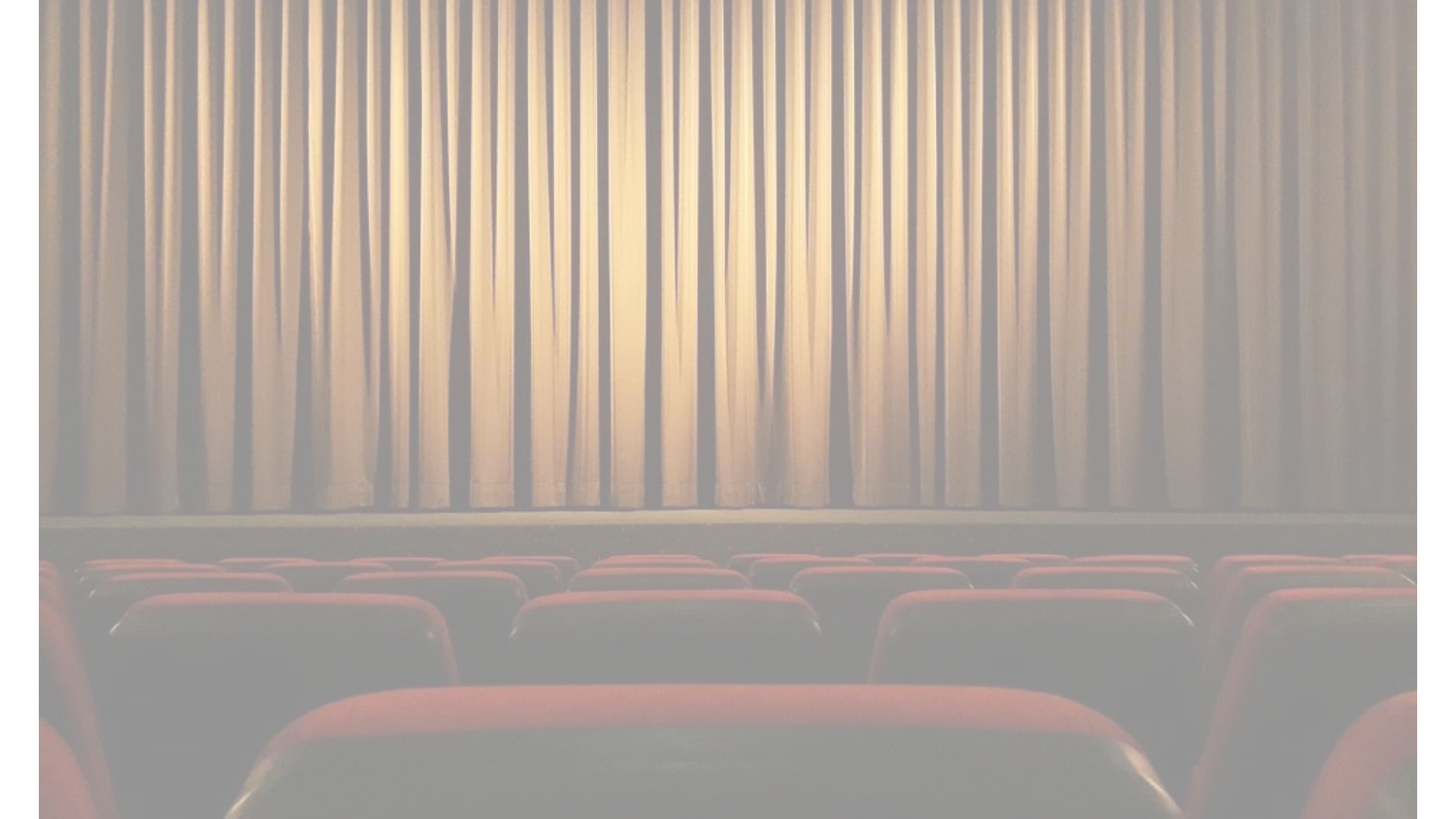

## Slide 12
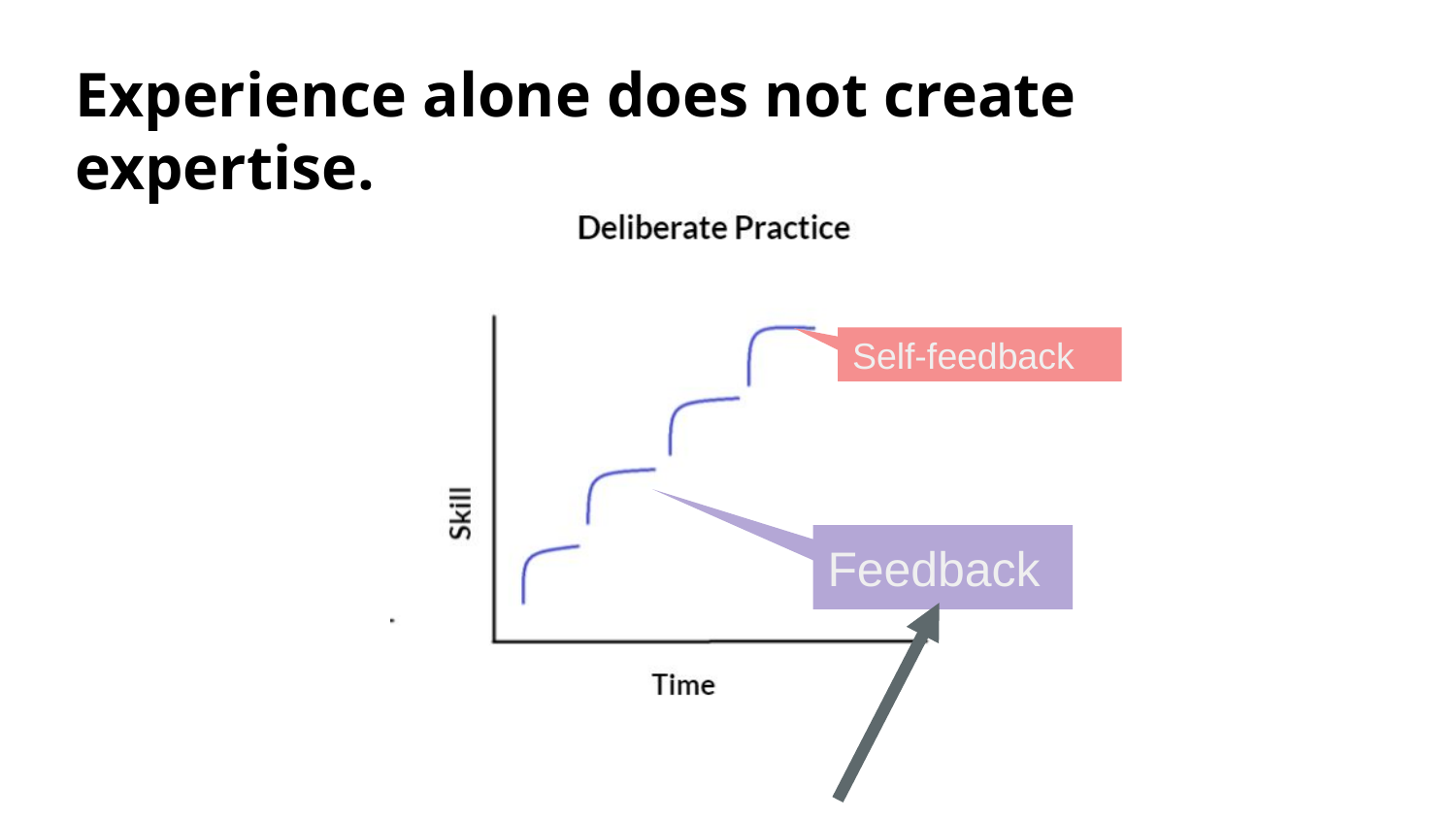

Experience alone does not create expertise.
Self-feedback
Feedback

## Slide 13
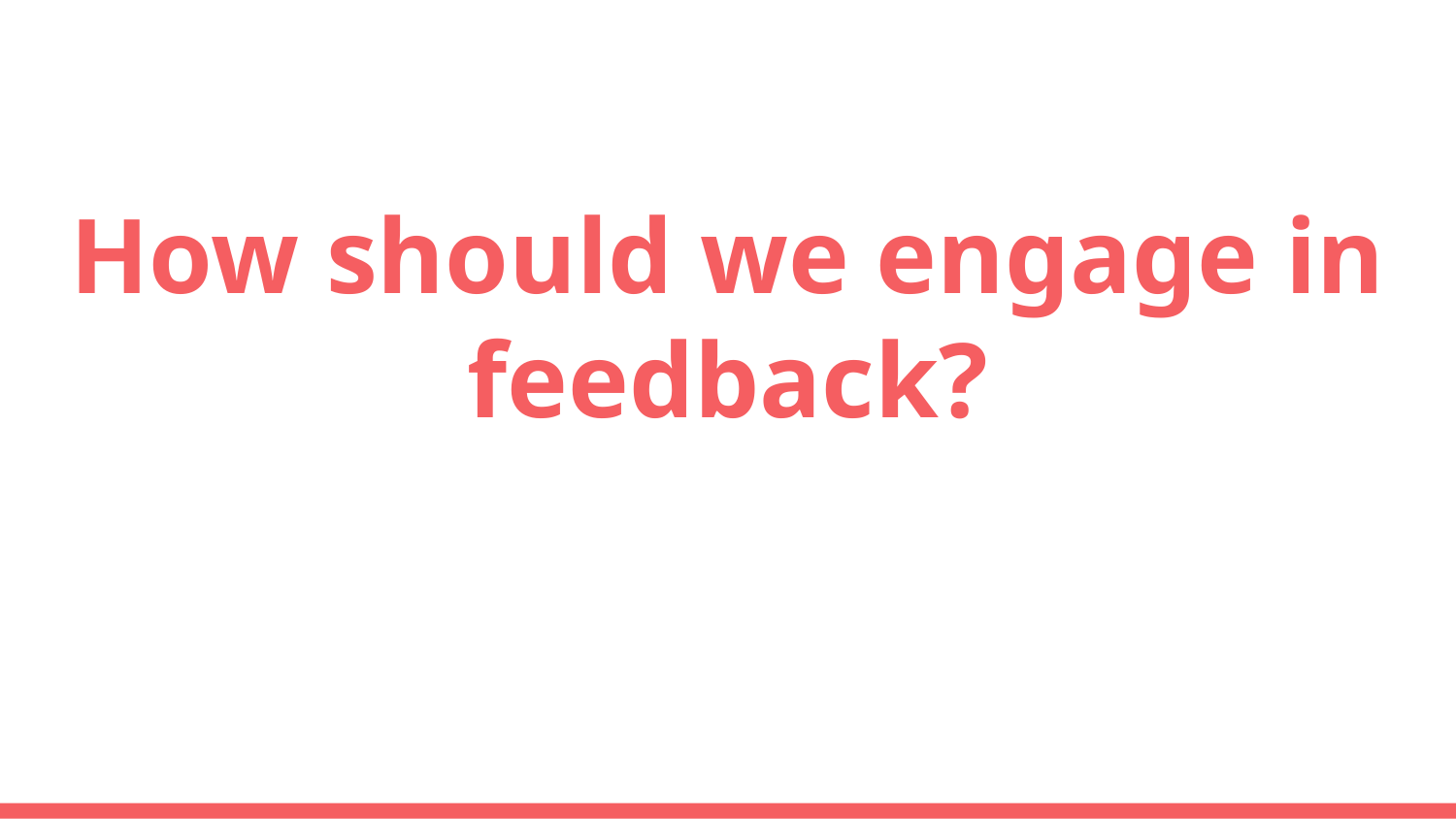

# How should we engage in feedback?

## Slide 14
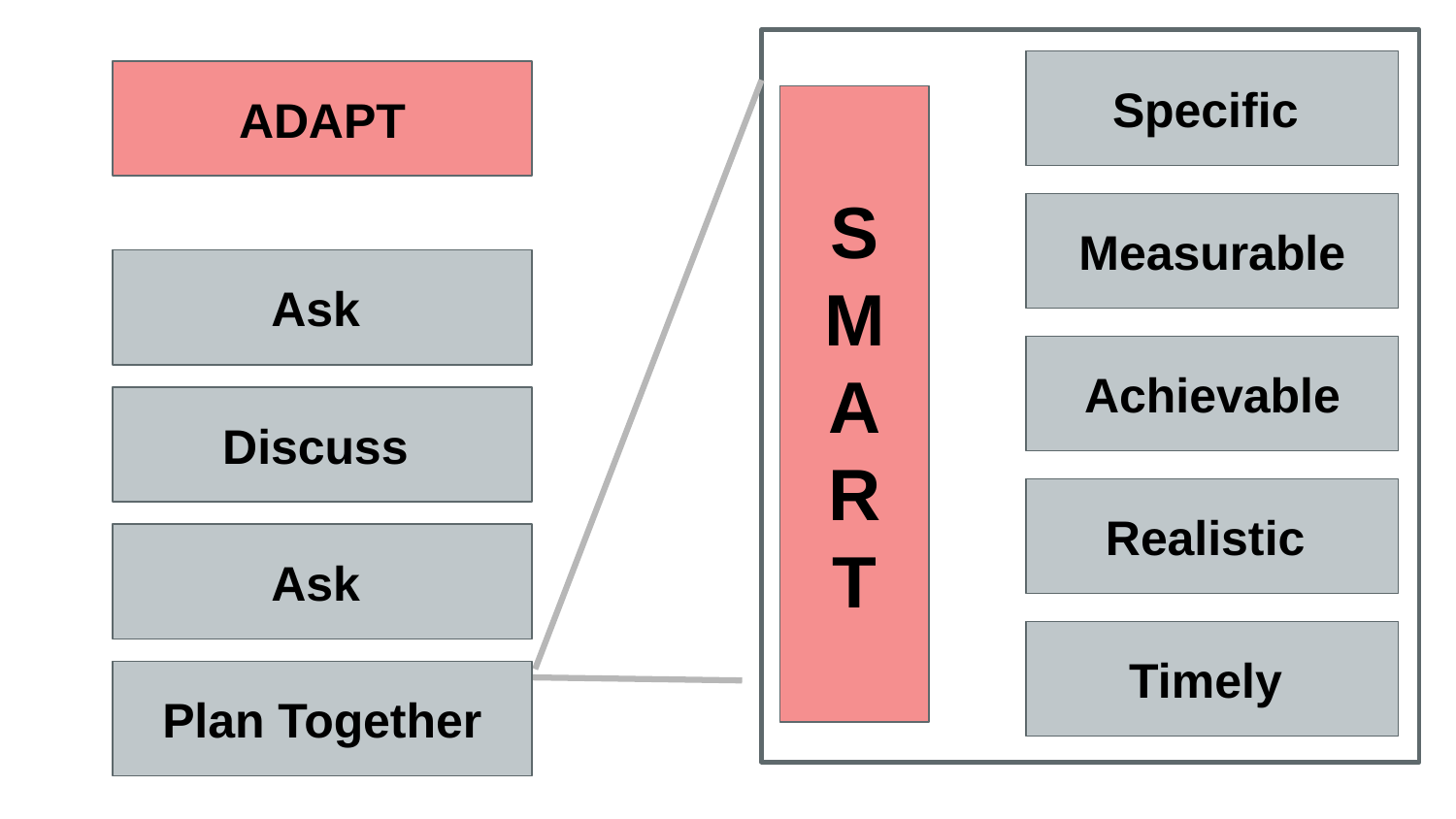

Specific
ADAPT
S
M
A
R
T
Measurable
Ask
Achievable
Discuss
Realistic
Ask
Timely
Plan Together

## Slide 15
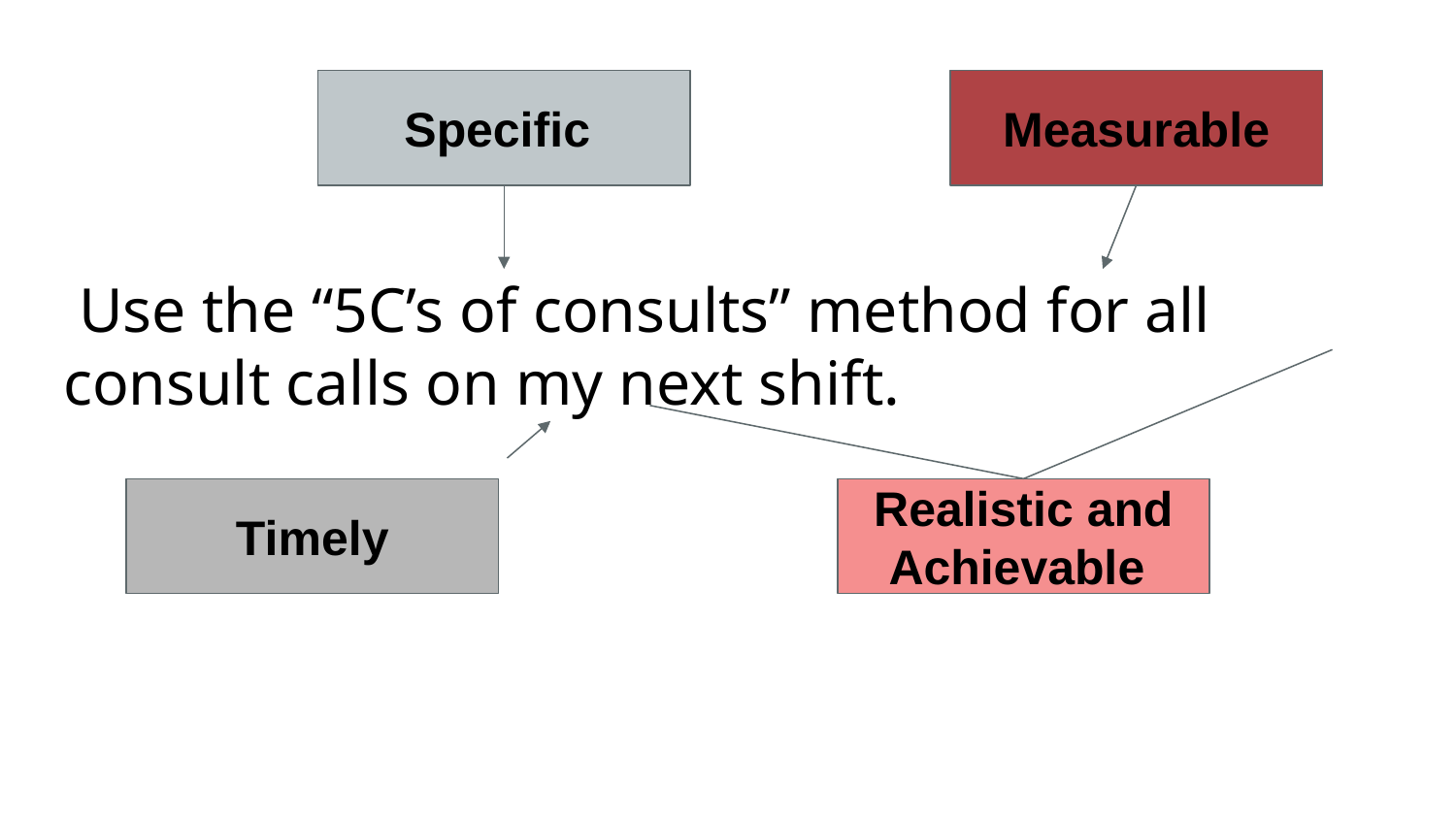

Specific
Measurable
 Use the “5C’s of consults” method for all consult calls on my next shift.
Timely
Realistic and Achievable

## Slide 16
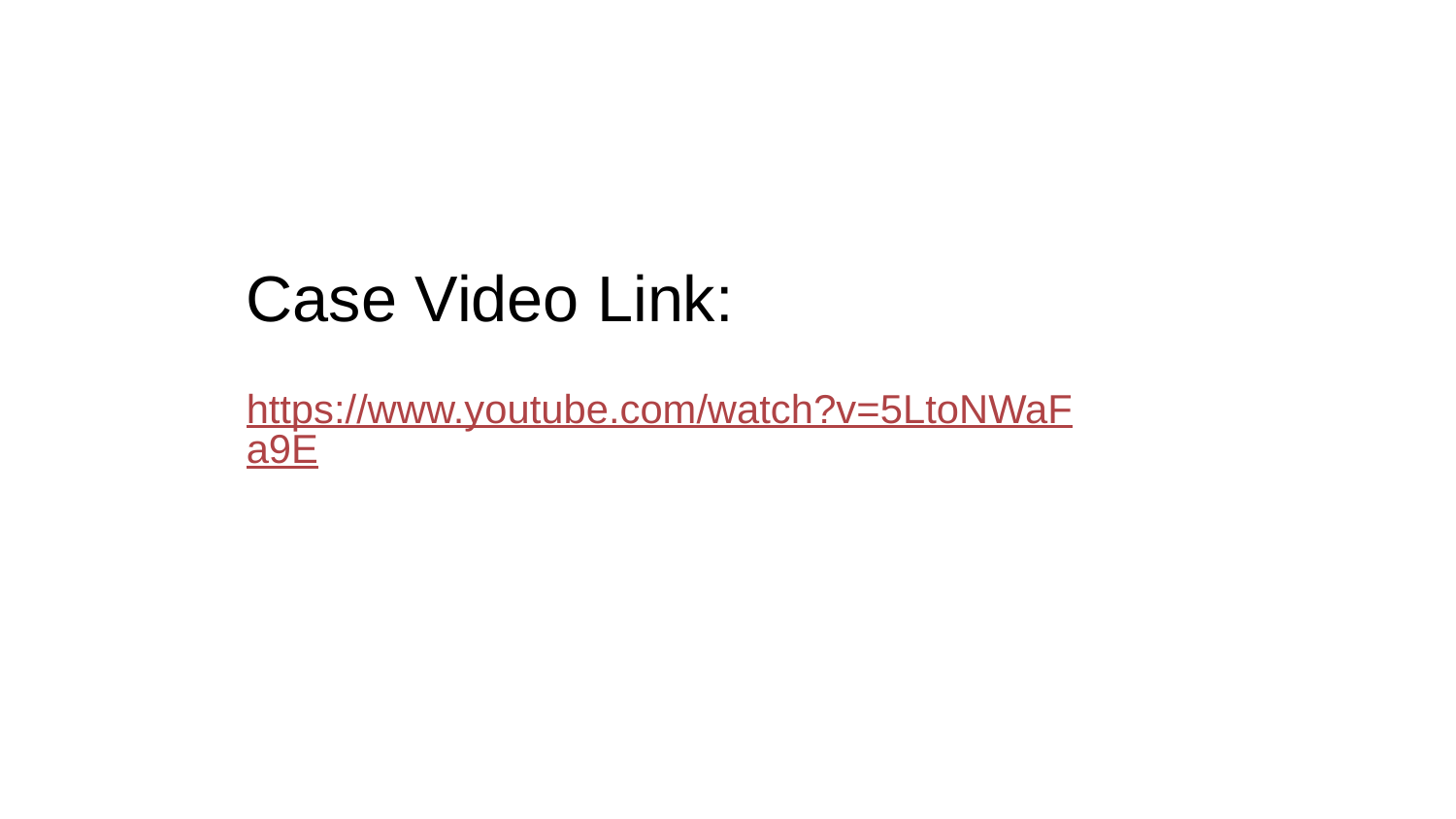

Case Video Link:
 https://www.youtube.com/watch?v=5LtoNWaFa9E

## Slide 17
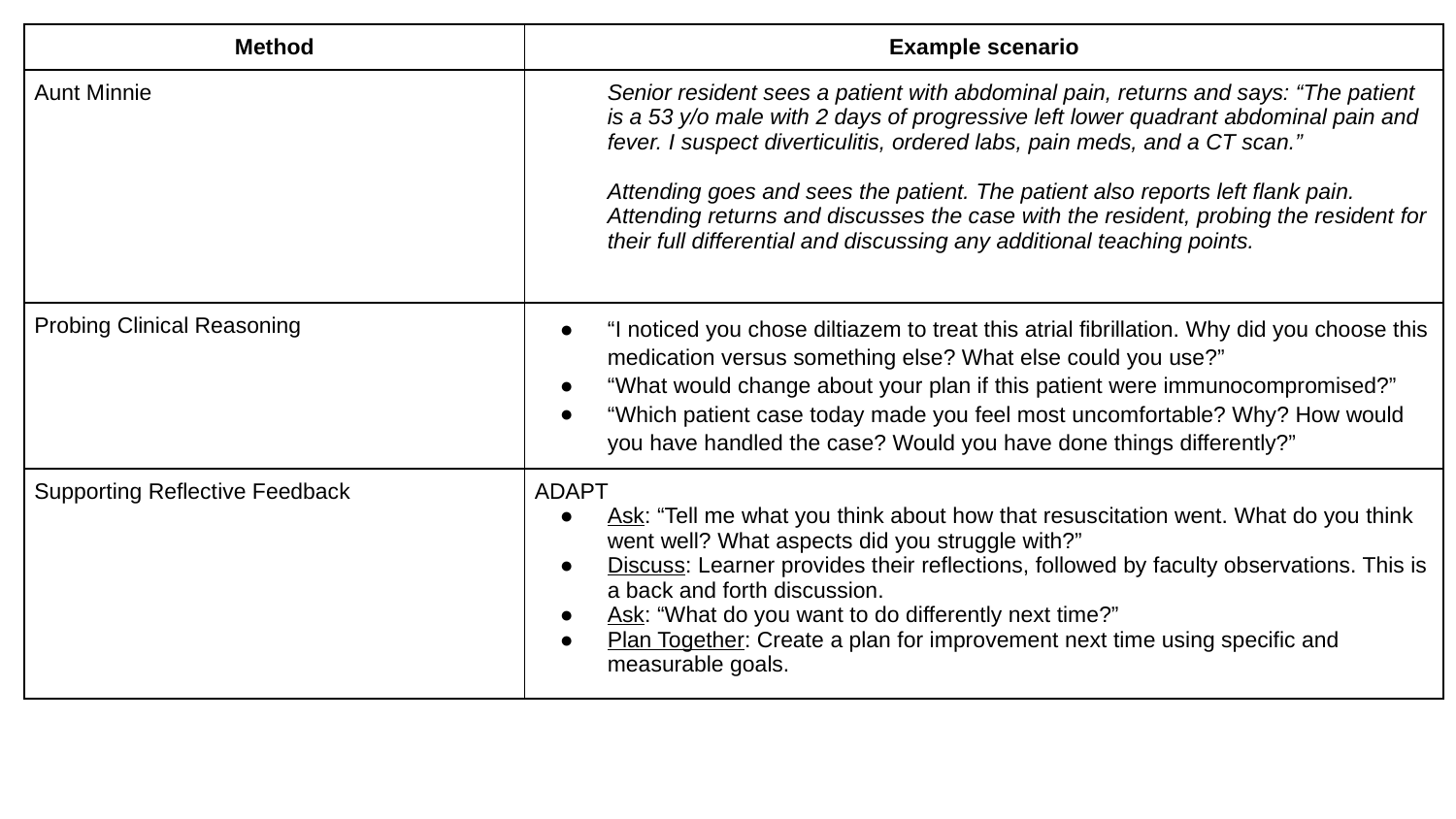

| Method | Example scenario |
| --- | --- |
| Aunt Minnie | Senior resident sees a patient with abdominal pain, returns and says: “The patient is a 53 y/o male with 2 days of progressive left lower quadrant abdominal pain and fever. I suspect diverticulitis, ordered labs, pain meds, and a CT scan.” Attending goes and sees the patient. The patient also reports left flank pain. Attending returns and discusses the case with the resident, probing the resident for their full differential and discussing any additional teaching points. |
| Probing Clinical Reasoning | “I noticed you chose diltiazem to treat this atrial fibrillation. Why did you choose this medication versus something else? What else could you use?” “What would change about your plan if this patient were immunocompromised?” “Which patient case today made you feel most uncomfortable? Why? How would you have handled the case? Would you have done things differently?” |
| Supporting Reflective Feedback | ADAPT Ask: “Tell me what you think about how that resuscitation went. What do you think went well? What aspects did you struggle with?” Discuss: Learner provides their reflections, followed by faculty observations. This is a back and forth discussion. Ask: “What do you want to do differently next time?” Plan Together: Create a plan for improvement next time using specific and measurable goals. |

## Slide 18
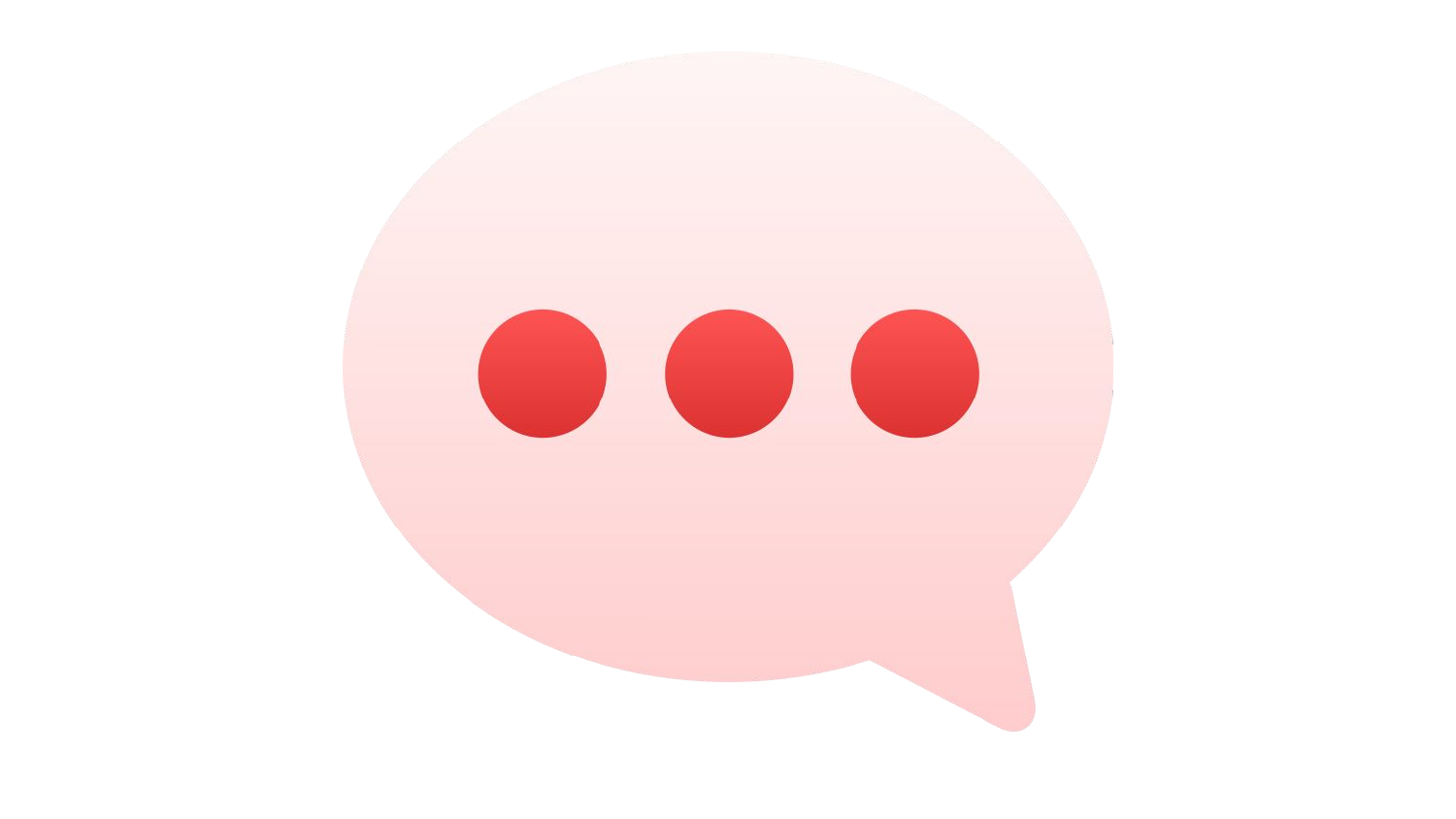

Supplement: Supplementary file 1 [file JETem-6-4-L7-supp1.pptx]
